# Excepta

**Published:** 1881-02

**Authors:** 


### EXCERPTA.

### A SIMPLE EXPLANATION.

Dr. John Brown, of Edingburg—he who introduced us all to dear Rab and his friends—tells the following anecdote: Walking through the grounds of a lunatic asylum one morning he was accosted by one of the inmates. "You don't know me," said the lunatic. "No," said Dr. Brown; "Who are you?" "I am Moses, the lawgiver," he replied. Expressing his pleasure at meeting the distinguished legislator, Dr. Brown continued his walk, and after a while he fell in with the lunatic again. "You don't know me," he said. "No," said Dr. Brown again; "Who are you?" "I am the Emperor Napoleon," he answered. "But," said Dr. B., "It was only fifteen minutes ago that you told me that you were Moses, the lawgiver." "Certainly," replied the lunatic; "*that was by another mother?*"—*Louisville Medical News.*

Bishops and Doctors.—I am not ashamed to say I have a son a doctor.—*Speech of the Bishop of Liverpool to medical men.*

How kind of the Bishop, and how patronizing,
And yet to his Punch 't is a little surprising
That, speaking to medical men there in session,
He dared speak of shame and a noble profession.
A bishop looks after our souls, but how odd is
The sneer that 's implied at the curers of bodies;
For surely it would be no hard task to fish up
A hundred brave doctors as good as the Bishop.

—*Punch*

### CURIOSITIES OF VITAL STATISTICS.

In a recent lecture the Rev. J. A. Nutting, of Fall River, Mass., quoted a series of vital statistics, to show the relationship between the birth rate and the number of divorces. He said:—

"During the past twenty-five years the birth rate has decreased about as fast as the divorce rate has increased, and where the birth rate is lowest—which is where yankees most abound—the divorce rate is highest. There is a close connection between a low birth rate and a high divorce rate," expressing his belief that "the relation between the two is that of cause and effect. In the history of nations there never has but three times occurred such a breaking up of the family as is now taking place among people of New England blood. When the Greek and Roman empires were about to fall, and during the French Revolution of the last century, twenty thousand divorces were obtained in France in one and a half years. Bad as this is, when population is compared, it is not equal to what is true of Rhode Island and Connecticut of late years.

Without vouching for the entire correctness of these statements, we believe they are largely true, and their cause is not far to seek.

To Terminate Chloroform Narcosis.—Schirmer (*Centralb. f. Augen.*) claims to induce rapid recovery by irritating the nasal mucous membrane. It has long been known that the fifth nerve retains its sensibility longer than any other part in narcosis, and that reflexes may be induced through it when other irritations fail. Schirmer uses simply a role of paper, which he turns in the nose. In bad cases he dips the paper in ammonia.

### BORAX IN HOARSENESS.

This salt has been employed with advantage in cases of hoarseness and aphonia occuring suddenly from the action of cold. The remedy is recommended to singers and orators whose voices suddenly become lost, but which by these means can be recovered almost instantly. A little piece of borax, the size of a pea, is to be slowly dissolved in the mouth ten minutes before singing or speaking. The remedy provokes an abundant secretion of saliva, which moistens the mouth and throat. This local action of the borax should be aided by an equal dose of nitrate of potassium, taken in warm solution before going to bed.—*La France Medicale.*

### PHYSIOLOGICAL ACTION OF CONIUM MACULATUM.

At a recent meeting of the Academie des Sciences (*Bull. Gen. de Therap.*, 1880, p. 356) M. Bouchefontaine alluded to the fact that in

1878 he had, in collaboration with M. Tiryakian, presented a paper on conium maculatum which went to show that hemlock owes its properties to two active constituents,—one, conine or cicutine, paralyzing the central nervous system, the other acting like curara. In 1879 M. Prevost, of Geneva, presented a note to the Academy, in which he considered the bromhydrate of cicutine as a paralyzant of the motor nerves.

Bouchefontaine, by recent experiments, has satisfied himself that conine diminishes or abolishes the physiological properties of the nervous centres before acting, like curara, on the nervo-muscular cement-substance (*substance jonctive*). On the dog and frog this alkaloid always ends by abolishing the nervous excito-mobility if it is given in sufficient quantity, but it is then fatal to batrachians and mammifers. Its action is therefore different from that of curara. The effects of the bromhydrates extracted from hemlock in a crystallized condition are as follows.

They are to be divided into two groups. One is composed of amber-colored crystals, is more toxic than the other, acts like conine, and represents the most active principle of hemlock. The other variety, of crystals, which are less poisonous, are colorless or of a pearly lustre, and resemble those obtained by Prevost. They act differently, however. As to the comparative action of hemlock and curara, this may be formulated thus: hemlock may act like curara, but it produces, in addition, certain physiological effects not observed in animals to whom curara has been administered.—*Phila. Med. Times.*

### CHRYSOPHANIC ACID IN SKIN-DISEASE.

Dr. J. Eagee Finny says (British Med. Journal:) There is no doubt of the superiority of chrysophanic acid over pyrogalic acid in the treatment of chronic psoriasis. To put the matter to a test, I have, in several instances of general psoriasis, directed one drug to be applied to one side of the patient's body, and the other on the opposite. This I conceived to be a test least liable to the fallacy to which the employment of the medications to different cases of psoriasis, or at different stages of it in the same individual, would be open. In all such test-cases, the parts where the chrysophanic acid was used, invariably recovered soonest. There are, doubtless, some cases in which great care must be taken, owing to its irritating properties on delicate skins, and the edema which it may produce when applied to the head and face ; but these reasons for caution do not in any way remove it from the list of the most efficient remedies for psoriasis. Where the epidemic scales are very thick, and where there is reason to believe that the acid is not acting, owing to the scales not being removed by soft soap prior to inunction, I have found the happiest results follow its use, after the removal of all scales down to the corium, by rubbing it firmly to the affected places, by means of a ball or lint, a six per cent solution of salicylic acid in rectified spirit—a line of treatment recommended by Dr. Priessman.

Dr. J. Marion Sims.—The Medical Public will be pained to learn that this distinguished physician has been desperately ill with pleuro-pneumonia. He is now convalescent or at least out of danger, though entire rest and perhaps change of climate will be found absolutely necessary for his perfect restoration.—*American Medical Bi-Weekly.*

### MEDICAL USES OF INDIAN HEMP.

Dr. Michael (*Montpellier Med.; Bull. Gen. de Therap.*; 1880, p. 380) again calls attention to the value of Indian hemp, particularly in uterine affections. He proposes the following formula in metrorrhagia:

| | | |
|---|---|---|
| ℞ | Tincturæ cannabis indicæ, . . . . | ʒ ss ; |
| | Syrupi simplicis, , . . . . . | f ℥ j ; |
| | Aquæ ad, . . . . . . . . | f ℥ viij.—M. |

Sig.—A teaspoonful every five or six hours.

His experience leads to the following conclusions: 1. The action of Indian hemp is double,—excitant in small doses, in larger ones sedative and even hypnotic. 2. Of use in most nervous affections, it is particulary valuable in chorea, tetanus, certain cases of mental alienation, delerium tremens and neuralgia. 3. The muscular tissue of the uterus is particulary sensitive to its influence; metrorrhagia is stopped by it and the uterine contraction so increased that it might be substituted for ergot.

### DECEM'R REPORT OF DEATHS AND INTERMENTS IN BALTIMORE.

| CAUSE OF DEATH. | No. | CAUSE OF DEATH. | No. | CAUSE OF DEATH. | No. |
|---|---|---|---|---|---|
| Abscess | 2 | Entero Chitis | 1 | Meningitis reflex | 1 |
| Albuminuria | 1 | Epilepsy | 2 | Necrosis | 1 |
| Aneurism of Aorta | 1 | Emphysema | 3 | Old Age | 22 |
| Angina Pectoris | 1 | Enlargement of liver | 1 | Operation of Hair Lip | 1 |
| Apoplexy | 10 | Exposure | 1 | Prog. locomoto ataxia | 1 |
| Asphyxia | 3 | Fever, malarial | 2 | Prostatitis | 2 |
| Asthma | 4 | " chagres | 1 | Paralysis | 14 |
| Asthenia | 7 | " puerperal | 4 | Pneumonia | 64 |
| Actelectasis Pulmonum | 1 | " remittent | 1 | " typhoid | 8 |
| Bright's Disease | 9 | " scarlet | 39 | " broncho | 1 |
| Burns | 8 | " typhoid | 17 | " pleuro | 1 |
| Capillary Bronchitis | 11 | " typho-malarial | 1 | Phthisis | 1 |
| Cancer, Diffused | 2 | Gangrene | 4 | Premature birth | 7 |
| " Breast | 1 | Gastro enteritis | 2 | Protracted labor | 1 |
| " Liver | 1 | Hernia, Strangulated | 1 | Purpura | 1 |
| " Face | 1 | Hemorrhage, bowels | 1 | Puerpural peritonitis | 1 |
| " Uterus | 2 | " lungs | 1 | Rheumatism, inflammatory | 3 |
| " Stomach | 2 | " uterus | 1 | " heart | 1 |
| Calculus | 1 | Hydrocephalus | 4 | Scrofula | 4 |
| Catarrh, senile | 1 | Inanition | 9 | Softening of brain | 3 |
| Cirrhosis of Liver | 1 | Inflammation of brain | 2 | Septicæmia | 1 |
| Congestive Chill | 1 | " bladder | 3 | Scalds | 2 |
| Congestion of Brain | 2 | " bowels | 5 | Sun stroke | 1 |
| " Lungs | 1 | " stomach | 2 | Stenosis | 1 |
| Consumption of Lungs | 91 | " bronchi | 10 | Syphilis | 2 |
| Convulsions | 19 | " kidneys | 4 | Thrombosis | 1 |
| " puerperal | 1 | " liver | 2 | Tetanus | 1 |
| Croup | 28 | " peritoneum | 3 | Trismus nascentium | 4 |
| Cyanosis | 4 | Intussusception | 1 | Ulcer, stomach | 1 |
| Dentition | 9 | Intemperance | 1 | " lungs | 1 |
| Diabetes | 2 | Myelitis | 1 | " bowels | 1 |
| Diphtheria | 36 | Malformation | 1 | Unknown, Adult | 1 |
| Disease of heart | 28 | Mal-nutrition | 3 | Unknown Infantile | 4 |
| " liver | 1 | Marasmus | 16 | Whooping Cough | 13 |
| Dropsy, (Gen'l) | 6 | Metro peritonitis | 1 | Wounds | 9 |
| " renal | 1 | Meningitis | 11 | Erysipelas | 4 |
| " heart | 1 | " Tubercular | 4 | | |
| Dysentery | 1 | " cerebro spinal | 2 | Total | 656 |

THE DEATHS REGISTERED IN THE MONTH INCLUDE

| | | | |
|---|---|---|---|
| Under 1 year | 152 | Between 20 and 30 " | 49 |
| Between 1 and 2 years | 61 | " 30 and 40 " | 42 |
| " 2 and 5 years | 53 | " 40 and 50 " | 41 |
| | | " 50 and 60 " | 56 |
| Under 5 years | 266 | " 60 and 70 " | 48 |
| Between 5 and 10 years | 56 | " 70 and 80 " | 40 |
| " 10 and 15 " | 13 | " 80 and 90 " | 19 |
| " 15 and 20 " | 22 | " 90 and 100 " | 4 |

NATIVITY, ETC.

| | |
|---|---|
| United States—White | 397 |
| " Colored | 149 |
| Foreign | 110 |
| Total for the month | 656 |
| Still Birth | 46 |

Among the decedents were 67 above the age of 70 years, whose combined ages were 5,241 years, averaging 78 years 3 month.

Estimated Population, 393,796. white, 338,384. Colored, 55,412. Annual Death Rate during the month, whole population, per 1,000—19.97. White, 17.94. Colored, 32.50.

For the year 1880, there were 13,260 marriages, 8,826 births, 8,043 deaths, 636 still births. Marriage rate per 1,000 population, 8.27. Births, 22.40. Deaths, 20.41. Still Births, 1.61. Death rate per 1,000, white population, 18.25. Colored, 34.05.

By Order: JAMES A. STEUART, M. D.,
Commissioner of Health and Registrar.

A. R. CARTER, Secretary.

U. S. SIGNAL OFFICE, BALTIMORE, MD., January 1, 1881.

### METEOROLOGICAL SUMMARY FOR THE MONTH OF DECEMBER, 1880.

### PRESSURE.

Mean Monthly Barometer, - - - - - 30.082 inches.
" " " for past 10 years, - - 30.131 "
Highest Barometer, - - - - - - 30.568 " on the 10th.
Lowest " - - - - - - - 29.672 " on the 5th.
Monthly Range of Barometer, - - - - 0.896 "

### TEMPERATURE.

Mean Monthly Thermometer, - - - - - 31.6 degrees.
" " " for past 10 years, - 36.4 "
Highest Thermometer, - 56 degrees on 5th. Monthly Range, - - 59 degrees.
Lowest " - - 3 " below zero on 30th. Mean Daily Range, - - 12.3 "

### WIND, ETC.

Prevailing Direction of wind, Northwest. Total Amount of Rainfall, 4.89 inches.
MeanVelocity of Wind, 6.2 miles per hour. Number of Days on which Rain fell, 18.
Highest Velocity of Wind, 22 " " on 7th. Mean Relative Humidity, 69.2 per cent.
Total Movement of Wind, 4,661 miles. ROBERT SEYBOTH, in charge.

